# Increased urinary excretion of kynurenic acid is associated with non-recovery from acute kidney injury in critically ill patients

**DOI:** 10.1186/s12882-018-0841-5

**Published:** 2018-02-26

**Authors:** Fabienne Aregger, Dominik E. Uehlinger, Gerhard Fusch, Aldin Bahonjic, Rene Pschowski, Michael Walter, Joerg C. Schefold

**Affiliations:** 1 Department of Nephrology and Intensive Care Medicine, Charité Universitätsmedizin Berlin, Campus Virchow Klinikum, Berlin, Germany; 2 Department of Nephrology and Hypertension, Inselspital, Bern University Hospital, University of Bern, Bern, Switzerland; 3 Department of Pediatrics, McMaster University, Hamilton, ON Canada; 4 Department of Gastroenterology, Charité Universitätsmedizin Berlin, Campus Virchow Klinikum, Berlin, Germany; 5 Institute of Laboratory Medicine, Clinical Chemistry and Pathobiochemistry, Charité University Medicine, Berlin, Germany; 6 Labor Berlin - Charité Vivantes Services GmbH, Berlin, Germany; 7 Department of Intensive Care Medicine, Inselspital, Bern University Hospital, University of Bern, 3010 Bern, Switzerland

**Keywords:** Intensive care unit, Renal failure, Inflammation, Tryptophan metablism, Kynurenines, IDO, Renal recovery

## Abstract

**Background:**

Acute kidney injury (AKI) is often observed in critically ill patients and is associated with high morbidity and mortality. Non-recovery from AKI has a negative impact on the prognosis of affected patients and early risk stratification seems key to improve clinical outcomes. We analyzed metabolites of a conserved key inflammatory pathway (i.e. tryptophan degradation pathway) in serial urine samples of patients with AKI.

**Methods:**

One hundred twelve ICU patients with AKI were included in a prospective observational analysis. After exclusion criteria, 92 patients were eligible for analysis. Serial urine samples were collected and tryptophan levels including key tryptophan metabolites were measured using tandem mass spectrometry.

**Results:**

Sixty-seven patients recovered in the first 7 days of AKI (early recovery, ER) whereas *n* = 25 had late−/non-recovery (LNR). Urinary concentrations of tryptophan, kynurenine, 3-OH anthranillic acid, serotonine, and kynurenine/tryptophan were significantly lower in LNR patients. In contrast, creatinine normalized excretion of kynurenic acid (KynA) was substantially increased in LNR patients (7.59 ± 6.81 vs. 3.19 ± 3.44 (ER) μmol/mmol, *p* <  0.005). High urinary KynA excretion was associated with higher RIFLE class, longer AKI duration, increased need for RRT, and 30-day mortality. Logistic regression revealed KynA as the single most important predictor of renal recovery on days 1 and 2 of AKI.

**Conclusions:**

Increased urinary levels of kynurenic acid, a key inflammatory metabolite of the tryprophan degradation pathway, are associated with adverse renal and clinical outcomes in critically ill patients with AKI. Urinary KynA may serve as an early risk stratificator in respective patients with AKI.

## Background

AKI (acute kidney injury) is common in critically ill patients in intensive care units and is associated with high morbidity and mortality [1, 2]. Development and validation of an AKI definition resulted in a huge progress in regard to the understanding of AKI in the last 12 years. Moreover, renal outcome analysis after survival from AKI has become an important field of scientific interest [3–6]. In addition, AKI is associated with an increased risk for chronic kidney disease [5] and non-recovery from AKI is associated with decreased long-term survival, indicating the importance of novel strategies designed to improve recovery from AKI [6]. Prediction of recovery from AKI seems therefore important to identify respective patients at increased risk. Recent studies focussed on renal recovery in AKI patients [7–10] and biomarkers with a special focus on inflammatory markers were investigated (e.g. NGAL [7], cytokines [8], inflammatory markers, hepatocyte growth factor, cystatin C and creatinine [9]). Using proteomics, we recently identified urinary insulin-like growth factor binding protein 7 (IGFBP-7) as a marker for prognosis in AKI [10].

Tryptophan (Trp) is an essential amino acid which undergoes degradation via the immunoregulatory enzyme indoleamine 2,3-dioxygenase (IDO) and, to a less extent, tryptophan 2,3 dioxygenase (TDO). The degradation of tryptophan along the IDO-pathway is a key and highly conserved inflammatory pathway that was previously shown important in a number of clinical conditions associated with acute and chronic inflammation, including renal allograft rejection [11] and chronic kidney disease (CKD) [12]. We previously demonstrated increased levels of IDO activity in patients with CKD and found a stepwise increase in Trp catabolite concentrations with advancing CKD class [12]. In fact, enzymatic degradation of tryptophan was shown to occur in numerous diseases such as stroke [13], severe infection [14, 15], hepatic dysfunction [16], and cardiac arrest [17, 18], which underlines the importance of the tryptophan degradation pathway as a key inflammatory pathway.

The rate of tryptophan degradation expressed by the ratio of substrate (tryptophan, trp) to metabolite (kynurenine, kyn) kyn/trp was reported to serve as an estimate of IDO activity [11, 12]. Moreover, the urinary Kyn/Trp ratio showed a strong dose-response relationship with incident myocardial coronary events, acute myocardial infarction, and mortality in a large cohort of stable patients undergoing coronary angiography [19] which may furthermore point to a significant clinical relevance of this inflammatory pathway.

The aim of the present study was to analyze urinary tryptophan excretion and levels of tryptophan metabolites in the urine of critically ill patients with AKI. We therefore analyzed serial urine samples collected during the first days of AKI. Urinary excretions were assessed in patients with early recovery (ER) and late−/non-recovery (LNR) from AKI using tandem mass spectrometry.

## Methods

The study was performed as a sub-investigation of a prospective proteomic AKI study [10]. Urine samples were prospectively collected from AKI patients at the medical ICU of the University Hospital Charité Campus Virchow-Klinikum, Berlin, and at the surgical ICU of the “German Heart Center Berlin” between 2010 and 2011. These patients corresponded to a “discovery group” which served as source for the proteomic approach. Inclusion criteria were: AKI (please note definition provided below) diagnosed in adults (age > 18 years) on the first day of AKI. Exclusion criteria were follow-up period of less than 7 days, presence of macrohematuria, end-stage renal disease, and renal transplantation.

### Study population and collection of urinary samples

**Fig. 1:**
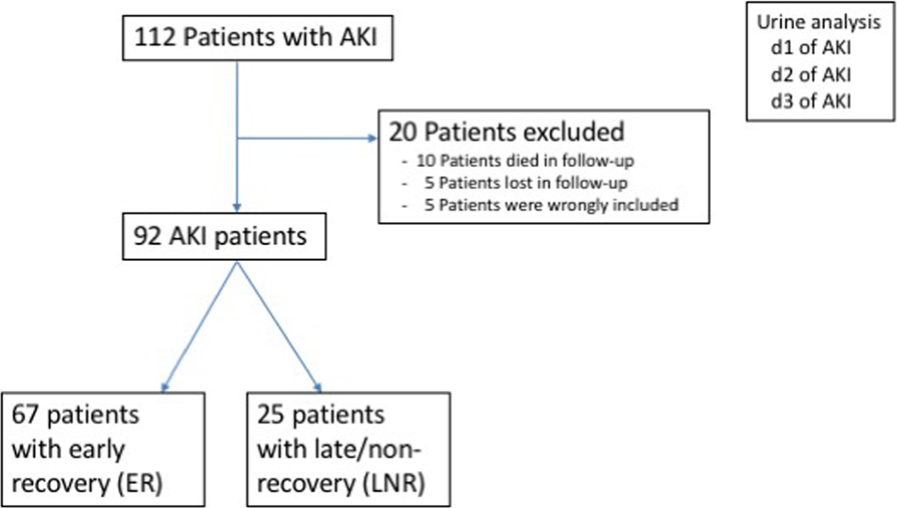
Study design. Urine was collected on the first 3 days in 112 acute kidney injury (AKI) patients on the intensive care unit. Urinary concentrations of tryptophan and respective metabolites were assessed on the first 3 days of AKI. After 1 week, recovery status was assessed. Results are compared between patients with early recovery (ER) and late−/non-recovery (LNR)

One hundred twelve AKI patients were enrolled in the study. Twenty patients were excluded due to death during follow-up, lost to follow-up, or wrongful inclusion (i.e. patients whose baseline creatinine concentration later turned out as increased and did thus not qualify for AKI according to the given definition). A study flow chart is given in Fig. 1. In total, 92 patients were eligible for analysis. Of these, 67 (73%) patients recovered during the first week after AKI (early recovery, ER) and *n* = 25 (27%) did not (late−/non-recovery, LNR). Aliquots of spot urine samples (drawn from respective catheters) were collected between 9 am and 11 am on the respective days of AKI, and were centrifuged, aliquoted and stored at − 80° until analysis. All samples were thawed only once prior to analysis.

### Definition of AKI

AKI was defined according to the RIFLE classification [20]. Only patients with a known baseline creatinine concentration were considered. Baseline creatinine was defined according to the method suggested by Pickering with one modification [21]: in patients hospitalized longer than 10 days on ICU, we deliberately defined baseline creatinine concentration as the lowest creatinine concentration during the ICU stay before development of AKI. GFR was estimated according to the Modification of Diet in Renal Disease (MDRD) equation. Recovery was defined as not classifying for any RIFLE class during the follow-up time of 7 days. Creatinine concentrations were determined daily.

### Assessment of tryptophan degradation products

Tryptophan metabolties were measured as previously reported [12, 22, 23]. In summary, Trp and respective metabolites were measured using tandem mass spectrometry in line with the procedure proposed by Zhu et al. [24]. For determination of creatinine levels, the method of Husková was modified to fit to the existing method [25]. Commercially available Trp, Kyn, KynA, quinolinic acid (QuinA), 5-hydroxy tryptophan (OH-Trp), 3-hydroxy anthranilic acid (3-OH-AA), serotonine, phenylalanine (all Sigma-Aldrich, St. Louis, USA) and creatinine (Cr) from Merck (Darmstadt, Germany), deuterium labelled compounds Kyn-d_6_, KynA-d_5_, Phe-d_5_, Trp-d_5_ (all Cambridge Isotope Laboratories, Andover, MA, USA), Cr-d_3._ (C/D/N Isotopes Inc., Pointe-Claire, QC, Canada), water (Optima MS grade, Fisher Scientific, Waltham, USA) and acetonitrile (Optima grade, Fisher Scientific, Waltham, USA) were used. One hundred microliters of urine were added to a deuterated internal standard (IS) mixture (50 μl) of equal volumes of Kyn-d_6_, Kyn A-d_5_, Phe-d_5_, Trp-d_5_ and Cr-d_3._ After shaking of the solution for 2 min, 500 μL acetonitrile was added and left over night at − 20 °C to precipitate the protein. Samples were centrifuged (20,000 *g*, 10 min), and the supernatant was dried under vacuum centrifugation (Savant SpeedVac Plus SC210A and Savant Refrigerated Vapor Trap RVT 4104). Dried samples were reconstituted with 100 μL H_2_O/acetonitrile (95%/5%). A Waters Acquity UPLC-TQD system (Milford, MA, USA) was equipped with an electrospray ion source using MRM detection in a positive ion mode. The following transitions of mass-to-charge ratios (*m/z*) of 205/188 for Trp, 210/193 for Trp-d_5_, 209/192 for Kyn, 215/198 for Kyn-d_6_, 168/150 for Quin, 190/144 for Kyna, 195/149 for Kyna-d_5_, 154/136 for 3HAA, 177/160 for Ser, 221/204 for OH-Trp, 166/120 for Phe, 171/125 for Phe-d_5_, 114/86 for Cr, and 117/89 for Cr-d_3_ were detected using argon as a collision gas. For separation of analytes, an Acquity UPLC BEH C18 column (1.7 μm, 100 mm) was used. A gradient of water/acetonitrile was utilized starting at a ratio of 97/3 and ramping up to a ratio of 70/30 in 5 min using a flow rate of 0.35 mL/min. Calibration curves were performed for quantification and referring the analytes to appropriate deuterated standards. Calibration curves were fitted by linear least-square regression. Serum (ClinChek- Control, Recipe, Germany) with known Phe and Trp concentrations was used as quality control to ensure the accuracy and precision of both the sample preparation and the measurements produced by the ultra performance liquid chromatography (UPLC)-MS/MS. Plasma creatinine concentrations were measured using a Roche-Cobas clinical chemistry autoanalyzer (Roche Diagnostics, Switzerland). In one NR patient estimated urinary creatinine concentrations were unrealistically low and negative on day 2. Urine samples of this individual patient were thus excluded from statistical analyses.

### Statistical analysis

Data are given as mean ± standard deviations. Nonparametric tests (Mann-Whitney U test) were done whenever appropriate. Correlation analysis were performed using Spearman’s rank correlation coefficient. Sensitivity, specificity, 95% CI, and AUC was calculated with GraphPad Prism v.7.0a (GraphPad Software, San Diego, CA). Univariate and multivariate logistic analyses, receiver operating characteristics and mixed effects models were performed using SAS 9.4 on a X64 VSPRO platform (SAS Institute, Cary, NC) using the procedures LOGISTIC, GLIMMIX and NLMIXED.

## Results

Samples from 92 patients with AKI were analysed (Fig. 1). Baseline characteristics of 67 patients with and 25 patients without early recovery are given (Table 1). Patients in both groups were comparable regarding comorbidities, and distribution of age and gender. Baseline disease severity as assessed using the APACHE II scoring system [26] and SOFA score on the first day of AKI was higher in LNR patients. Furthermore, haemoglobin concentrations and thrombocyte counts on day 1 of AKI were lower in LNR patients. Of note, creatinine concentrations on the first day of AKI did not differ between the two groups (*p* = 0.2). As expected, renal function during the follow-up time of 7 days was reduced in LNR patients (Table 2) and respective patients required RRT therapy more often, received more transfusions, had a longer hospital and ICU-stay, and had a higher 30-day mortality.

**Table 1:** Baseline characteristics

	Early recovery	Late/Non-Recovery	*p*
Number	67	25	
Age, years (mean ± SD)	68 ± 14	65 ± 15	NS
Female gender, n (%)	19 (28)	11 (44)	NS
Hypertension, n (%)	43 (64)	15 (60)	NS
Diabetes, n (%)	21 (31)	6 (24)	NS
Baseline creatinine level, mg/dl (mean ± SD)	0.97 ± 0.38	0.98 ± 0.41	NS
Chronic kidney disease^a^, n (%)	19 (28)	5 (20)	NS
ICU admission due to			
Cardiovascular disease, n (%)	18 (27)	5 (20)	NS
Infection, n (%)	10 (15)	6 (24)	NS
Neurological disease	5 (7)	2 (8)	NS
Liver failure	1 (1)	3 (12)	NS
Kidney failure	2 (3)	1 (4)	NS
Surgery	31 (46)	8 (32)	NS
Time from ICU admission to day 1, days (median (IQR))	1 (IQR1-3)	1 (IQR 1-12)	NS
Patients with SIRS on day 1, n (%)	42 (63)	19 (76)	NS
Patient with SIRS and infection on day 1, n (%)	6 (9)	6 (24)	NS
APACHE 2 score on admission, (mean ± SD)	27 ± 7	32 ± 7	< 0.005
SOFA score^b^ on day 1, (mean ± SD)	9 ± 4	12 ± 4	< 0.005
Patients under vasoactive drugs, n (%)	42 (63)	18 (72)	NS
Invasive ventilation, n (%)	47 (70)	20 (80)	NS
Creatinine on day 1, mg/dl (mean ± SD)	0.97 ± 0.38	0.98 ± 0.41	NS
Oliguria^c^ on day 1, n (%)	7 (10)	3 (12)	NS
Hemoglobin on day 1, g/dl (mean ± SD)	108 ± 16	99 ± 20	< 0.05
Thrombocyte count on day 1, x 109/l (mean ± SD)	188 ± 98	130 ± 88	< 0.05
C reactive protein on day 1, mg/l (mean ± SD)	14 ± 10	12 ± 11	NS

^a^Chronic kidney disease defined as eGFR< 60 ml/min (MDRD formula), ^b^SOFA score without renal SOFA score, ^c^Oliguria defined as urinary output < 500 ml per day

**Table 2:** Clinical outcomes of study patients

	Early Recovery	Late/Non-Recovery	*p*-value
Number	67	25	
Maximum creatinine level, mg/dl (mean ± SD)	2.0 ± 0.9	3.6 ± 2.0	< 0.0001
Minimal eGFR, ml/min (mean ± SD)	45 ± 25	21 ± 9	< 0.0001
RIFLE class, R/I/F	42/18/7	0/6/19	< 0.0001
Duration of AKI, days (mean ± SD)	3.0 ± 1.8	21.5 ± 19.8	< 0.0001
Patients on RRT, n (%)	2 (3)	15 (63)	< 0.0001
Patients with RBC transfusions, n (%)	30 (45)	19 (76)	0.01
Red blood cell transfusions, n (mean ± SD)	1.4 ± 2.3	3.7 ± 4.1	< 0.001
Length of hospital stay, days (mean ± SD)	34 ± 33	50 ± 31	< 0.005
Length of ICU stay, days (mean ± SD)	18 ± 20	31 ± 26	< 0.01
28-day mortality, n (%)	13 (20)	11 (46)	< 0.05
90-day mortality, n (%)	25 (34)	15 (63)	NS

*AKI* acute kidney injury, *eGFR* estimated glomerular filtration rate, *ICU* intensive care unit, *RBC* red blood cellm, *R* Risk, *I* Injury, *F* Failure, *RRT* renal replacement therapy

### Urinary excretion of tryptophan metabolites

**Fig. 2:**
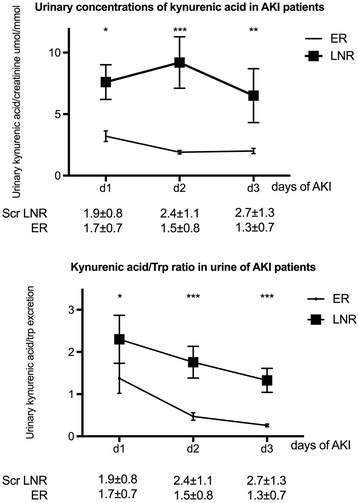
Urinary concentrations of kynurenic acid and kynurenic acid/tryptophan ratio in AKI patients with/without early recovery. Results are given on a linear scale and displayed as boxplot summaries. The middle line in the box represents the mean, and the whiskers represent the standard error of the mean. Urinary concentrations of creatinine normalized kynurenic acid (KynA) were increased in patients with late−/non-recovery (LNR) when compared to patients with early recovery (ER) (*p ≤ 0.005, **p < 0.001 and ***p < 0.0001). The urinary ratio of KynA/tryptophan was higher in LNR patients compared to ER patients. (*p = 0.01, *** < 0.0001). Corresponding serum creatinine concentrations in mg/dl are given below the graph

On day 1 of AKI, urinary creatinine normalized concentrations of Trp, Kyn, 3-OH-Anthranilic acid (3-OH-AA), serotonine, phenylalanine, OH-Trp and Kyn/Trp were not different between the two groups (Table 3). Creatinine normalized urinary concentrations of KynA (ER: 3.19 ± 3.44 μmol/mmol vs. LNR: 7.59 ± 6.81 μmol/mmol creatinine, *p* <  0.005) and the ratio of KynA to Trp (ER: 1.37 ± 2.86 μmol/mmol vs. LNR: 2.30 ± 2.73 μmol/mmol, *p* = 0.01) were higher in LNR patients (Table 3, Fig. 2). Increased concentrations of KynA in LNR patients were consistently observed on days 2 and 3 of AKI (Fig. 2). The same applied to the ratio of KynA/Trp. On day 3, creatinine normalized urinary concentrations of Trp, Kyn, 3OH-AA and serotonine in LNR patients were lower as compared with ER patients and so was the ratio of Kyn/Trp.

**Table 3:** Urinary concentrations of Tryptophan and respective metabolites

		DAY 1			DAY 2			DAY 3		
		ER	LNR	*p*	ER	LNR	*p*	ER	LNR	*p*
Trp/cr	umol/mmol	5.51 ± 4.95	6.10 ± 6.75	NS	9.50 ± 14.91	7.28 ± 7.84	NS	12.08 ± 10.49	6.0 ± 5.6	< 0.005
Kyn/cr	umol/mmol	2.90 ± 4.87	2.94 ± 7.65	NS	3.88 ± 5.69	4.30 ± 8.80	NS	5.60 ± 8.17	2.2 ± 4.8	< 0.0001
KynA/cr	umol/mmol	3.19 ± 3.44	7.59 ± 6.81	< 0.005	1.87 ± 1.10	9.17 ± 9.67	< 0.0001	2.00 ± 1.28	6.51 ± 9.49	< 0.001
3 OH AA/cr	umol/mmol	1.36 ± 2.86	1.04 ± 1.82	NS	1.14 ± 1.62	0.72 ± 1.04	NS	2.02 ± 3.11	0.75 ± 0.99	< 0.05
Serotonine/cr	umol/mmol	0.045 ± 0.075	0.053 ± 0.11	NS	0.038 ± 0.026	0.036 ± 0.041	NS	0.045 ± 0.035	0.027 ± 0.037	< 0.005
Phenylalanine/cr	umol/mmol	5.50 ± 5.02	9.41 ± 12.84	NS	7.56 ± 11.80	9.77 ± 12.03	NS	8.49 ± 7.55	6.73 ± 6.81	NS
OH-Trp/cr	umol/mmol	0.12 ± 0.18	0.23 ± 0.72	NS	0.11 ± 0.077	0.13 ± 0.106	NS	0.11 ± 0.068	0.11 ± 0.078	NS
KynA/Trp		1.37 ± 2.86	2.30 ± 2.73	< 0.01	0.47 ± 0.66	1.8 ± 1.8	< 0.0001	0.26 ± 0.20	1.33 ± 1.25	< 0.0001
Kyn/Trp		0.66 ± 1.24	0.34 ± 0.33	NS	0.64 ± 1.08	0.58 ± 0.93	NS	0.73 ± 1.58	0.30 ± 0.41	< 0.05

*3OH AA* 3-hydroxy anthranilic acid, *cr* creatinine, *d* day, *ER* early recovery, *Kyn* kynurenine, *KynA* kynurenic acid, *LNR* late−/non-recovery, *OH-Trp* 5-hydroxy tryptophan, *Trp* tryptophan. Urinary concentrations of Trp and respective metabolites were normalized to urinary creatinine concentrations (except KynA/Trp ratio). Results are given as mean ± standard deviations for the first 3 days of AKI (d1, d2, and d3)

### Prognostic performance of tryptophan metabolites

Logistic regression analysis revealed KynA as the single most important predictor of renal recovery on days one and two of AKI (score Х^2^ = 12.7, *p* <  0.0004, and Х^2^ = 20.6, *p* <  0.0001, respectively). On AKI day 3, the single most important predictor of renal recovery was serum creatinine (Х^2^ = 17.3, *p* < 0.0001). Using multivariate logistic analysis with stepwise selection from SOFA score at admission, serum creatinine, eGFR, diuresis, KynA and the ratio of KynA to Trp revealed the following best models to predict outcome: day 1: KynA (Х^2^ = 12.7, *p* < 0.0004) and SOFA score at admission (Х^2^ = 6.1, *p* < 0.0134), day 2: KynA (Х^2^ = 20.6, *p* < 0.0001) and serum creatinine (Х^2^ = 8,4, *p* < 0.0039) and day 3: serum creatinine (Х^2^ = 17.3, *p* < 0.0001) and the ratio of KynA to Trp (Х^2^ = 7.6, *p* < 0.0059). Combining the data of all 3 days into one logistic mixed effects model revealed no significant improvement when the ratio of KynA to Trp as compared to KynA was modelled together with the SOFA score at admission and serum creatinine levels (Akaike information criterion AIC 103.6 vs. 102.5). Potential predictors of early recovery on day 1 of AKI are given in Table 4.

**Table 4:** Potential early predictors of renal recovery

	*ROC AUC*	*CI 95%*	*Wald χ2*	*P<*
KynA	0.72	0.59-0.85	8.88	0.003
SOFA score	0.70	0.67-0.83	6.75	0.01
Delta diuresis (day 1 – baseline), ml/h	0.68	0.55-0.81	3.84	0.05
Diuresis, ml/h	0.63	0.51-0.76	3.58	0.06
Delta serum creatinine (day 1 – baseline)	0.61	0.47-0.74	2.14	0.14
Serum creatinine, mg/dL	0.59	0.46-0.72	1.38	0.24

All data based on the initial values collected on day one

*KynA* kynurenic acid, *SOFA* sepsis-related organ failure assessment score

**Fig. 3:**
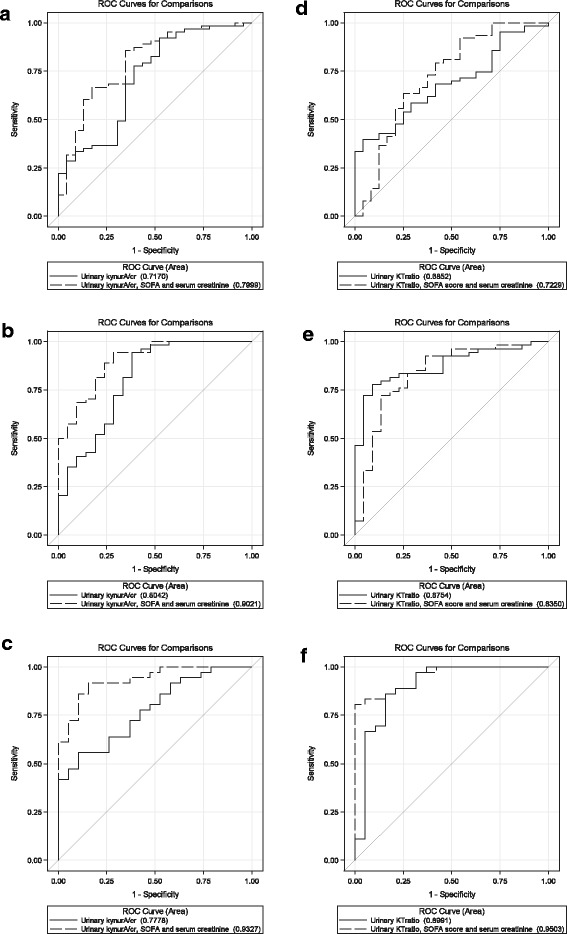
Multivariate logistic regression model of renal recovery in AKI patients. ROC analyses for the prediction of renal recovery by urinary KynA (**a**-**c**) and the ratio of urinary KynurA/cr to tryptophan/cr (**d**-**f**), respectively; alone (full line) and in combination with the clinical parameters (SOFA score at admission and serum creatinine levels, dashed line) at days 1 (**a**, **d**), 2 (**b**, **e**) and 3 (**c**, **f**) of AKI

ROC analyses of KynA revealed an AUC for predicting renal recovery of 0.72 for KynA (95%- CI: 0.59-0.85, *p* < 0.003) on day 1, 0.80 (95%-CI 0.69-0.93, *p* < 0.003) on day 2 and 0.78 (95%-CI 0.66-0.90, *p* < 0.023) on day 3 (Fig. 3a–c). When SOFA scores at admission and serum creatinine concentrations were included into the model, the prognostic power improved to an AUC of 0.93 (95%-CI 0.87-1.00, *p* < 0.011) on day 3. AUC of KynA/Trp to predict recovery revealed 0.69 (95%-CI 0.56-0.79, NS) on day 1, 0.88 (95% CI 0.80-0.96, *p* < 0.002) on day 2 and 0.90 (95% CI 0.80-1.00, *p* < 0.001) on day 3, respectively (Fig. 3d–f). When SOFA score and serum creatinine concentration were included into the model, AUC further improved and revealed 0.95 (95%-CI 0.90-1.00) on day 3. AUCs on day 1 for Trp and all other metabolites did not reach statistical significance (data not shown).

### Correlation analysis of Trp metabolites

We observed a negative correlation of urinary KynA excretion on day 1 with eGFR (*r* = − 0.24, *p* < 0.05) and for KynA/Trp with eGFR (*r* = − 0.51, *p* < 0.0001). KynA and the ratio of KynA/Trp on day one correlated with AKI duration (*r* = 0.4, *p* < 0.0001 and *r* = 0.39, *p* < 0.0005, respectively), the need for renal replacement therapy (*r* = 0.39, *p* < 0.0005 and 0.30, *p* < 0.005 respectively), and non-recovery (*r* = 0,35, *p* < 0.001 and *r* = 0.27, *p* < 0.01 respectively). KynA was furthermore found to correlate with 30- and 90-day mortality (*r* = 0.28, *p* < 0.001 and *r* = 0.25, *p* < 0.005, respectively).

## Discussion

After analysis of serial urine samples from 92 AKI patients, we found that urinary concentrations of KynA and the ratio of KynA/Trp predict renal recovery on day 1 of AKI. Prognostic performance furthermore improved when key clinical characteristics such as SOFA score and serum creatinine concentrations were included into the analysis. Logistic regression analysis consistently revealed KynA as the single most important predictor of renal recovery on days 1 and 2 of AKI. High urinary KynA excretion was associated with higher RIFLE class, longer AKI duration, increased need for RRT, renal non-recovery, and 30-day mortality.

Patients without recovery from AKI had advanced renal dysfunction and higher need for renal replacement therapy, received an increased amount of transfusions, and had both longer ICU- and hospital stay and increased mortality. This finding is in line with previous data demonstrating that non-recovery from AKI is associated with decreased short-term and long-term outcomes [6, 27, 28]. Until now, there was no clear definition of recovery from AKI and several autors suggested that a standardized definition of renal recovery might improve our understanding of factors associated with recovery [29, 30]. Very recently, the ADQI group proposed new definitions to define the course of disease after AKI [31]. In this consensus report, the group proposed new definitions reflecting the concept of a continuum of ongoing kidney injury. The group proposed AKI as a kidney injury lasting for 7 days, whereas acute kidney disease (AKD) was proposed as an acute renal damage for a duration between 7 and 90 days after exposure to an AKI initiating event. CKD was defined as persistent kidney disease 3 months after AKI. Even though we designed our study long before, hazard definitions may be regarded similar to the recent ADQI group consensus definitions. If we would apply the definition suggested by the ADQI group to our patients, we would compare AKI (early recovery) versus AKD (late/non-recovery) patients. Our data therefore demonstrate that patients with AKD have worse renal and non-renal outcome and that these patients can be recognized as early as day 1 of renal damage when analyzing tryptophan metabolites.

Reduced urinary excretion of Trp and most metabolites as well as high urinary concentrations of KynA were associated with poor clinical outcome. This seems in line with our previous data demonstrating a stepwise increase of plasma concentrations of Kyn, KynA, Serotonine and increased IDO activity in patients with progressive CKD while Trp plasma concentrations remain stable [12]. In line with our previous results, other groups analyzed Trp and respective metabolites in a rat model of CKD [32]. Plasma concentrations of Trp decreased slightly in severe CKD, whereas plasma concentrations increased slightly for Kynurenine and strongly for KynA (increase from 49 to 395 nM). Increased concentrations of KynA in the serum of uremic patients was reported more than 30 years ago [33] and the EUTox proposed KynA as an uremic toxin [34]. Furthermore, the ratio of plasma KynA/Trp was shown to be a sensitive marker for renal function in hypertensive patients with CKD [35]. Of interest, using liquid chromatography/mass spectrometry-based metabolite profiling, several markers including Kyn and KynA were identified in patients from the Framingham study developing CKD at a later stage [36]. In a subgroup of these patients, arterial and renal vein sampling was performed and V/A ratios were calculated (0.84 for creatinine, 0.59 for KynA and 0.7 for Kyn). This may suggest net uptake by the kidney via filtration, secretion, and/−or renal metabolism/catabolism. The same group further analyzed urinary excretion of these metabolites and showed fractional excretion of KynA of 180% suggesting tubular KynA excretion possibly via the human organic anion transporter OAT1 and/or OAT3 [37]. In our study, urinary excretion of KynA was negatively correlated with renal function. However, although the current observational study is unable to elucidate the underlying pathomechanisms involved in excretion of KynA, one might speculate that increase activation of inflammatory pathways resulting increased production of KynA in AKI, increased glomerular filtration, and increased release of KnyA from apoptotic/necrotic sells may explain the effects observed here.

Previously, urinary concentrations of Trp and its metabolites were not extensively studied. Urinary concentrations of KynA were reported to range between 4 and 13 umol/l in patients with normal renal function [38, 39]. Importantly, urinary concentrations of KynA in our AKI patients were substantially higher. Assuming a daily creatinine excretion of 10 mmol/day in our patients, daily excretion of KynA would be around 32-76 umol. Except for KynA, the urinary excretion of Trp and its metabolites decreased with declined renal function. This might partialy explain at least the superiority of the ratio of KynA to Trp over KynA excretion with ongoing renal failure, i.e. on days 2 and 3 of AKI. There are concerns about whether urinary AKI biomarkers should be normalized to urine creatinine [40, 41]. The underlying assumption that creatinine excretion is stable is flawed since there are dynamic changes in urine creatinine excretion during AKI. However, in several clinical studies, normalization of urinary biomarkers to urinary creatinine concentration improved AKI prediction [42]. We observed sligthly better performance of creatinine normalized KynA to predict recovery when compared to absolute concentrations (data not shown).

Our study has several limitations. First, patients were analyzed in a single center and samples were analyzed in a retrospective fashion despite prospective collection of samples [10]. Second, urine aliquots analyzed in the present study were stored for a longer period of time at − 80°. As this condition is identical for all aliquots, only a systematic error might apply. Third, although preferable, we are unable to provide data of disease-matched control patients as they were not available for the current analysis. Fourth, even though we did not observe differences in the distribution of cardiovascular or inflammatory disease between the groups, we cannot rule out a potential bias introduced by such comorbidities. Fifth, nutritional intake of tryptophan and/or tryptophan metabolites was not specifically investigated in this study. Although short-term intake might be of minor importance during the first days of critically illness, we are unable to rule out an effect of nutritional intake on our findings. Moreover, we observed associations rather than underlying mechanisms in this analysis as elucidating underlying pathomechanisms was beyond the scope of the current analysis. Last, we excluded 20 patients from analysis of which 10 died in the follow-up of time of 7 days. The reason for exclusion of these 10 patients was the impossibility to assess renal recovery due to preceeding death. Moreover, patients without known baseline creatinine were excluded, which may have theoretically biased our observational data. Although the general approach seems justifiable in a study on biomarkers to predict renal recovery, patients with poorest prognosis remain unstudied.

## Conclusions

In conclusion, we observed that increased urinary excretion of kynurenic acid measured on day 1 of AKI predicts renal non-recovery. High urinary kynurenic acid concentrations correlated with higher RIFLE class, longer AKI duration, increased need for RRT, increased rates of renal non-recovery, and increased mortality. Assessment of urinary KynA might therefore serve as a potential early risk stratificator in critically ill patients with AKI. Our data may add to a better risk stratification of affected patients in the future.
